# *Clostridioides difficile* Infection Among Hospitalized Patients With Cancer

**DOI:** 10.1001/jamanetworkopen.2026.2103

**Published:** 2026-03-25

**Authors:** Giovanni A. Roldan, Spencer Goble, Timothy Davie, Jesse Fletcher, Leticia Campoverde, María Alejandra Mendoza, Daphne M. Moutsoglou

**Affiliations:** 1 Division of Gastroenterology, Hepatology and Nutrition, University of Minnesota, Minneapolis; 2 Division of Hematology- Oncology, University of Texas, MD Anderson, Houston; 3 Division of Infectious Diseases, University of Utah, Salt Lake City; 4 Gastroenterology Section, Minneapolis Veterans Affairs Medical Center, Minneapolis, Minnesota; 5 Department of Medicine, University of Minnesota, Minneapolis

## Abstract

**Importance:**

Patients with cancer face an increased risk of *Clostridioides difficile* infection (CDI) due to several factors, including chemotherapy and repeated health care exposures. However, recent national-level data characterizing the prevalence of CDI, associated clinical outcomes, and resource utilization in this population remain limited.

**Objective:**

To characterize the hospitalization-level prevalence, clinical outcomes, and health care resource utilization associated with CDI among hospitalized patients with cancer in the US.

**Design, Setting, and Participants:**

This cross-sectional study used data from the National Inpatient Sample, a nationally representative database, to identify hospitalizations among adults with a diagnosis of cancer between January 1, 2016, and December 31, 2022. Hospitalizations with CDI were identified using *International Statistical Classification of Diseases and Related Health Problems, Tenth Revision* codes. Data were analyzed from May to June 2025.

**Exposures:**

Presence of CDI as a primary or secondary diagnosis during the index hospitalization.

**Main Outcomes and Measures:**

The primary outcome was in-hospital, all-cause mortality. Secondary outcomes included need for kidney replacement therapy, mechanical ventilation, vasopressor support, and colonoscopy. Multivariable logistic regression was used to adjust for demographics, comorbidities, cancer type, and hospital characteristics.

**Results:**

Of 32 083 671 cancer-related hospitalizations (overall study population mean [SD] patient age, 69.4 [13.9] years; 16 050 025 [50.0%] male), 450 360 (1.4%) involved a diagnosis of CDI. Compared with hospitalizations without CDI, those with CDI involved older patients, a higher proportion of women, and higher prevalence of hematologic cancers, cirrhosis, solid organ transplant, bone marrow transplant, chronic kidney disease, and inflammatory bowel disease. Patients with CDI infection had higher in-hospital mortality (7.3% vs 4.5%; adjusted odds ratio [aOR], 1.62; 95% CI, 1.58-1.67) and greater use of critical care interventions. CDI was associated with higher rates of critical care interventions, including kidey replacement therapy (44.3 vs 20.4 per 1000 hospitalizations; aOR, 2.00; 95% CI, 1.92-2.08; *P* < .001), mechanical ventilation (68.1 vs 35.6 per 1000 hospitalizations; aOR, 1.89; 95% CI, 1.84-1.95; *P* < .001), and vasopressor use (25.6 vs 11.5 per 1000 hospitalizations; aOR, 2.11; 95% CI, 2.00-2.24; *P* < .001). Mortality among patients with CDI varied geographically, ranging from 6.4% (95% CI, 6.2%-6.5%) in the Midwest to 8.5% (95% CI, 8.3%-8.7%) in the Northeast.

**Conclusions and Relevance:**

In this cross-sectional study of US oncologic hospitalizations, CDI occurred in 1 in 70 cancer-related hospitalizations and was associated with significantly increased mortality and resource utilization. These findings underscore the need for targeted prevention and early intervention strategies in this vulnerable population.

## Key Points

**Question:**

What are the hospitalization-level prevalence and associated clinical outcomes of *Clostridioides difficile* infection (CDI) among cancer-related admissions in the US?

**Findings:**

In this cross-sectional study of over 32 million cancer-related hospitalizations, 1.4% involved a diagnosis of CDI. Patients with CDI had higher in-hospital mortality (7.3% vs 4.5%) and greater use of critical care interventions. CDI was independently associated with a 62% increase in adjusted odds of mortality.

**Meaning:**

These findings suggest CDI is associated with adverse outcomes among hospitalized patients with cancer and may warrant targeted prevention strategies across high-risk malignant tumor types.

## Teaser

This cross-sectional study evaluates the hospitalization-level prevalence, clinical outcomes, and health care resource utilization associated with *Clostridioides difficile *infection among hospitalized patients with cancer in the US.

## Introduction

*Clostridioides difficile* infection (CDI) is one of the leading causes of health care-associated infection in the US, posing a significant public health burden in hospitalized patients and health care systems.^1,2,3^ In 2017, the Centers for Disease Control and Prevention estimated 293 300 new cases of hospital-acquired CDI nationwide.^4^ CDI is associated with recurrent hospitalizations, colectomy, concomitant infections, prolonged hospital stays, and mortality rates ranging from 6.9% to 16.7%.^4^ Additionally, CDI has been estimated to contribute $1.2 to $5.9 billion in annual health care expenditures in the US (in 2012 US dollars).^5^ Notably, hospital-acquired CDI accounts for 64.7% of total cases, and an additional 82% of the community-associated CDI cases are linked to outpatient health care exposure.^4^

Patients with cancer represent a uniquely vulnerable population for CDI due to both host-related and treatment-related conditions. Chemotherapy-induced neutropenia, frequent use of antibiotics, and prolonged or recurrent admissions contribute to impaired host defenses and increased risk of CDI.^4,6,7,8,9,10^ Additionally, some patients, particularly those with hematologic cancers such as leukemia, may experience profound cytopenia and immune dysfunction as a direct result of their disease, further increasing susceptibility to CDI. Several studies have demonstrated increased rates of CDI in this population, with estimates suggesting that they are nearly twice as likely to develop hospital-acquired CDI compared with patients without malignant neoplasms.^11,12,13^

Despite the increased recognition of the elevated risk of CDI in patients with cancer, recent population-level data evaluating the prevalence and hospitalization outcomes associated with CDI among cancer-related admissions in the US remain limited. Prior studies have primarily used institutional data, specific malignant tumor types, or narrow inpatient outcome measures.^12,13,14,15,16^ Although prior studies have used the National Inpatient Sample (NIS), the largest publicly available inpatient database in the US, to examine CDI in certain cancer populations, recent and comprehensive national data evaluating the prevalence and clinical outcomes of CDI across all hospitalized patients with cancer remain limited.^17,18^

Therefore, this study aimed to estimate the hospitalization-level CDI prevalence among cancer-related admissions in the US. We further aimed to characterize demographic features and evaluate clinical outcomes—including mortality, length of stay (LOS), number of procedures, and hospitalization charges—among hospitalized patients with cancer with and without CDI. By providing contemporary, nationally representative data, this study aimed to characterize the hospitalization-level prevalence and clinical associations of CDI in the cancer population, informing future efforts to improve prevention and outcomes in this high-risk group.

## Methods

### Study Design and Database Description

We performed a retrospective, cross-sectional analysis of the NIS from 2016 to 2022 that was conducted and reported in accordance with the Strengthening the Reporting of Observational Studies in Epidemiology (STROBE) reporting guideline for observational research. The NIS was developed as part of the Healthcare Cost and Utilization Project (HCUP) and represents the largest all-payer database within the US and is intended to approximate the total US inpatient population. The NIS characterizes approximately 20% of US admissions through a stratified, systematic sampling design. HCUP provides standard weighting procedures to convert results into national estimates. Weighting was used for all assessments in this study. The NIS includes information for admissions, including patient demographic data, clinical diagnoses, procedures, hospital characteristics, and health care utilization outcomes. The NIS is a publicly available, deidentified database so institutional review board approval and patient informed consent was waived in accordance with 45 CFR §46.

### Study Sample and Variables

We identified all adult hospitalizations within the NIS from 2016 to 2022 with a primary or secondary diagnosis of a malignant neoplasm based on the presence of relevant *International Statistical Classification of Diseases and Related Health Problems, Tenth Revision (ICD-10)* codes as previously demonstrated by Haider et al.^19^ Within this cohort, the presence or absence of CDI was then determined for each hospitalization based on *ICD-10* codes recorded during the index admission. Cancer and CDI were identified using *ICD-10* codes listed as either a primary or secondary diagnosis, which in the NIS represent the principal reason for admission or clinically relevant coexisting conditions present at admission or arising during hospitalization (eTable 1 in Supplement 1). Hospitalizations coded only with a history of cancer were not included, ensuring our cohort reflects patients with active malignant neoplasms.

Baseline demographic and clinical characteristics were compared between those with and without a diagnosis of CDI. Race and ethnicity were obtained from the NIS database, where they are reported by participating hospitals and reflect hospital administrative coding. The NIS does not specify whether race and ethnicity are self-reported or assigned. Race and ethnicity were included as covariates because they are available in the NIS and have been associated with differences in health care utilization and in-hospital outcomes. Malignant tumors were classified into the following categories: hematologic; ear, nose, and throat (ENT); gastrointestinal; respiratory; skin; breast; genitourinary and reproductive; endocrine; and other. Cancers within the other subgroup included secondary neoplasms, connective tissue tumors, neuroendocrine tumors, brain and spinal cord malignant tumors, hematopoietic neoplasms not elsewhere classified, and unspecified thoracic or ill-defined neoplasms (eTable 1 in Supplement 1). The skin cancer group included melanoma and nonmelanoma skin cancers.

### Outcomes

The primary objective was to describe hospitalization-level CDI prevalence and assess clinical outcomes associated with CDI among patients hospitalized with active malignant tumors. The following clinical outcomes were assessed: all-cause in-hospital mortality, kidney replacement therapy (KRT), mechanical ventilation, and vasopressor support (eTable 2 in Supplement 1). All-cause in-hospital mortality is an included variable within NIS, while KRT, mechanical ventilation, and vasopressor support were determined by the presence of relevant *ICD-10* procedure codes. In addition to comparing clinical outcomes in those with and without CDI, subgroup analyses were performed to compare outcomes by cancer subtype in the context of CDI.

The secondary objective was to evaluate the association between CDI and health care utilization in hospitalized patients with malignant tumors. The health care utilization outcomes included LOS (in days), total hospitalization cost (in US dollars), and cost per day. LOS is directly reported in the NIS, while hospitalization charges were converted to costs using the HCUP-provided cost-to-charge ratios. Cost per day was generated by dividing the total cost of a hospitalization by LOS.

### Statistical Analysis

Continuous variables were summarized as means with SDs and compared using *t* tests. Given the large sample size, this approach aligns with parametric assumptions and is consistent with prior NIS-based studies. Categorical variables were reported as proportions and were compared using χ^2^ tests. Differences in clinical outcomes between CDI and non-CDI hospitalizations are reported as odds ratios (OR) and adjusted odds ratios (aOR). CIs were reported at the 95% level. Multivariable logistic regression analysis was used to compare clinical outcomes. To account for skewed data, LOS was compared using negative binomial regression, while cost and cost per day were compared using generalized linear models.

The following covariates were included in all performed multivariable assessments: age, sex, race, income quartile, Charlson Comorbidity index, malignant tumor type, primary payer, hospital bed size, and hospital region. Hospitalizations with missing LOS data were excluded from the LOS assessments, and those with missing cost data were excluded from cost analyses. If either LOS or cost was missing, the patient was excluded from cost per day assessments (eTable 3 in Supplement 1). However, patients excluded from cost and LOS analysis were still included in clinical outcome assessments. Additionally, LOS analysis was restricted to patients who survived to discharge. Terminal hospitalizations were included in cost analysis.

All statistical analyses were performed using STATA version 17.0 (StataCorp). Figures were generated using RStudio, version 2024.12.1 + 563 (Posit Software). Data were analyzed from May to June 2025.

## Results

### Baseline Clinical Characteristics and Hospitalization-Level Prevalence of CDI

Among 32 083 671 cancer-related hospitalizations from 2016 to 2022 (mean [SD] age, 69.4 [13.9] years; 16 050 025 [50.0%] male; 3 718 630 [11.6%] Black, 2 371 379 [7.4] Hispanic, and 23 444 563 [73.1%] White), 450 360 hospitalizations (1.4%) had a primary or secondary diagnosis of CDI. CDI was more common among older patients, women, and those with chronic kidney disease (115 180 patients with CDI [25.6%] vs 5 876 949 patients without [18.2%]), inflammatory bowel disease (13 200 patients with CDI [2.9%] vs 311 165 patients without [1.0%]), and hematologic cancers (79 805 patients with CDI [21.8%] vs 3 315 539 patients without [13.1%]) (Table). CDI rates decreased with each subsequent year measured in the study (eTable 4 in Supplement 1).

**Table.** Patient Demographics With Cancer-Related Hospitalizations Stratified by the Presence of *Clostridioides difficile* Infection (CDI)

Variable	Patients, No. (%)		*P* value
	CDI (n = 450 360)	No CDI (n = 31 633 311)	
Age, mean (SD), y	69.6 (13.9)	69.4 (13.9)	<.001
Sex			
Female	240 945 (53.5)	15 784 496 (49.5)	<.001
Male	209 270 (46.5)	15 840 755 (50.5)	<.001
Race and ethnicity			
White	338 705 (77.3)	23 105 858 (75.6)	<.001
Black	46 575 (10.6)	3 672 055 (11.7)	<.001
Hispanic	30 890 (7.1)	2 340 489 (7.4)	<.001
Asian or Pacific Islander	9930 (2.3)	804 075 (2.6)	<.001
American Indian	1910 (0.4)	125 625 (0.4)	.10
Other^a^	10 295 (2.4)	769 804 (2.4)	.20
US hospital region			
Northeast	90 105 (20.0)	6 483 552 (20.0)	.86
Midwest	119 250 (26.5)	7 769 420 (24.0)	<.001
South	155 190 (34.5)	11 393 680 (37.4)	<.001
West	85 815 (19.1)	5 986 659 (18.5)	.01
Charlson Comorbidity Index, mean (SD)	4.2 (2.7)	3.8 (2.7)	<.001
Human immunodeficiency virus	3880 (0.9)	213 610 (0.7)	<.001
Chronic kidney disease	115 180 (25.6)	5 876 949 (18.2)	<.001
Cirrhosis	16 770 (3.7)	944 365 (2.9)	<.001
Solid organ transplant history	5930 (1.3)	212 365 (0.7)	<.001
Bone marrow transplant	3245 (0.7)	103 175 (0.3)	<.001
Crohn disease	4700 (1.0)	176 590 (0.6)	<.001
Ulcerative colitis	8500 (1.9)	134 575 (0.4)	<.001
Type of malignant neoplasm			
Hematologic	79 805 (21.8)	3 315 539 (13.1)	<.001
Ear, nose, and throat	4045 (1.7)	331 315 (1.9)	<.001
Gastrointestinal	52 335 (20.6)	3 597 954 (19.5)	<.001
Respiratory	21 240 (9.3)	2 005 174 (11.9)	<.001
Skin	30 325 (9.2)	2 887 274 (11.9)	<.001
Breast	46 985 (13.9)	3 863 659 (15.4)	<.001
Genitourinary and reproductive	115 430 (25.6)	9 218 892 (28.5)	<.001
Endocrine	7295 (2.2)	697 800 (2.8)	<.001
Other	106 620 (23.7)	7 574 208 (23.4)	.10

^a^ Individual hospitals provide data on patient races to the National Inpatient Sample without specifying if the data are self-reported. Other race is defined as any race not listed or multiple races being reported.

### Clinical Outcomes

**Figure 1.**
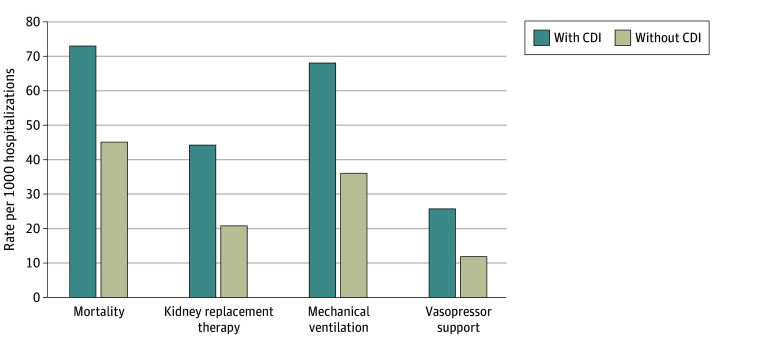
Bar Chart of Association Between *Clostridioides difficile* Infection (CDI) and Clinical Outcomes Among Hospitalized Patients With Cancer Rates (per 1000 hospitalizations) of mortality, kidney replacement therapy, mechanical ventilation, and vasopressor support stratified by presence or absence of *Clostridioides difficile* infection.

**Figure 2.**
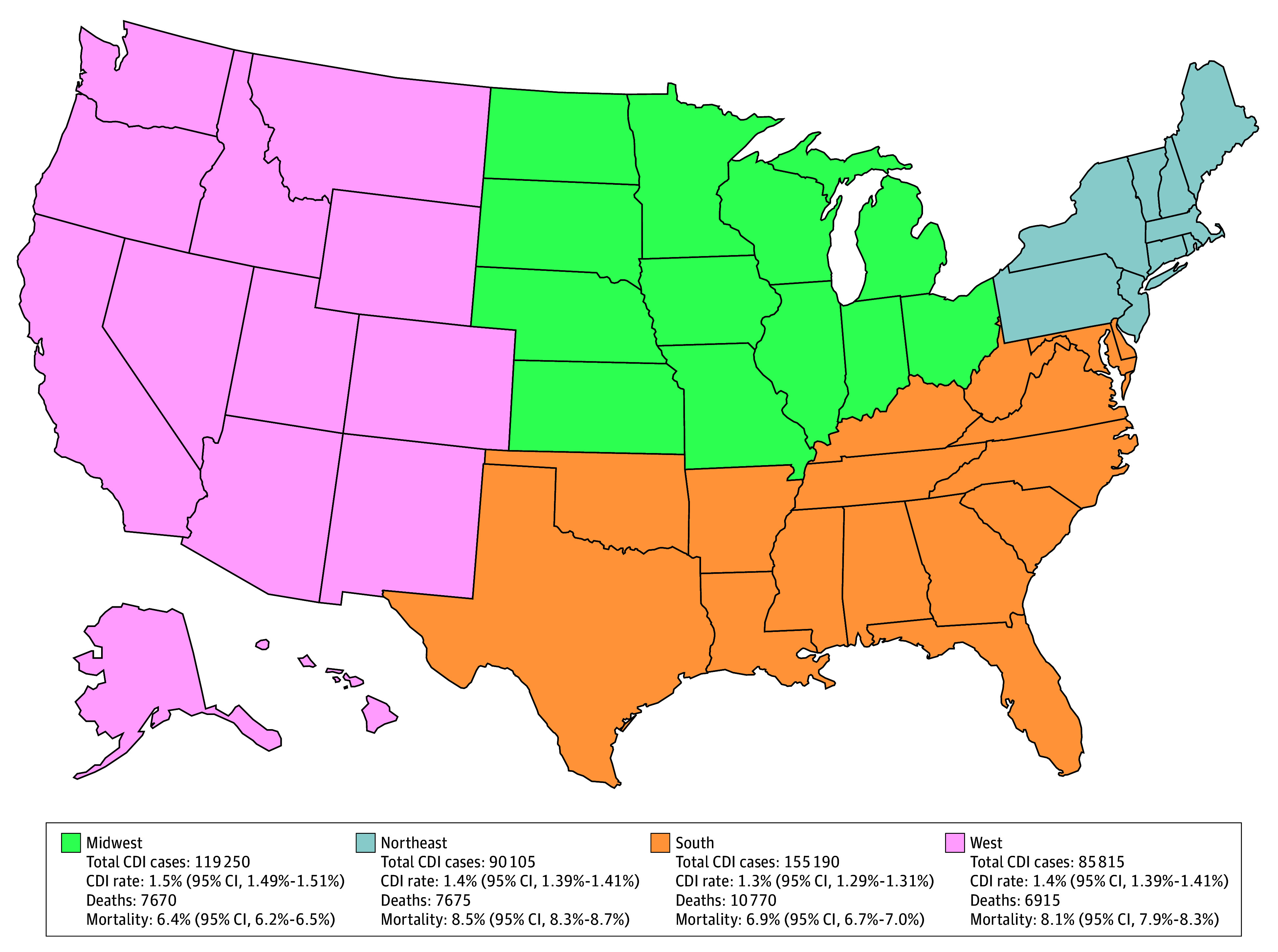
Map of Regional Prevalence and Mortality Associated With* Clostridioides difficile *Infection (CDI) Among Hospitalized Patients With Cancer in the United States, 2016 to 2022

CDI was independently associated with worse clinical outcomes among hospitalized patients with cancer. A total of 1 447 145 deaths were recorded, corresponding to an overall in-hospital mortality of 4.5%. When stratified by CDI status, hospitalized patients with cancer and CDI experienced significantly higher rates of adverse outcomes compared with those without CDI. In-hospital mortality was 73.3 versus 44.7 per 1000 hospitalizations (aOR, 1.62; 95% CI, 1.58-1.67) (Figure 1). Similarly, kidney replacement therapy was required in 44.3 versus 20.4 per 1000 hospitalizations (aOR, 2.00; 95% CI, 1.92-2.08), mechanical ventilation in 68.1 versus 35.6 per 1000 hospitalizations (aOR, 1.89; 95% CI, 1.84-1.95), and vasopressor support in 25.6 versus 11.5 per 1000 hospitalizations (aOR, 2.11; 95% CI, 2.00-2.24). Regional variation in CDI rates and outcomes was observed, with all-cause mortality rates ranging from 6.4% (95% CI, 6.2%-6.5%) in the Midwest to 8.5% (95% CI, 8.3%-8.7%) in the Northeast (Figure 2).

### Healthcare Utilization Outcomes

Among the 30 635 626 cancer-related hospitalizations with available data on LOS, the mean (SD) duration was 5.5 (6.7) days. Patients with CDI had a longer mean (SD) stay (10.4 [12.3] days) compared with those without CDI (5.6 [6.5] days). Similarly, among 31 837 041 hospitalizations with cost data, the mean (SD) hospitalization cost was $17 805 ($25 447) overall, with a higher mean (SD) cost observed in hospitalizations involving CDI ($28 753 [$55 005]) compared with those without CDI ($17 653 [$$24 754]). Interestingly, the mean (SD) daily cost of hospitalization was lower in patients with CDI ($2689 [$2120]) compared with those without CDI ($4172 [$4787])

### Subgroup Analyses

**Figure 3.**
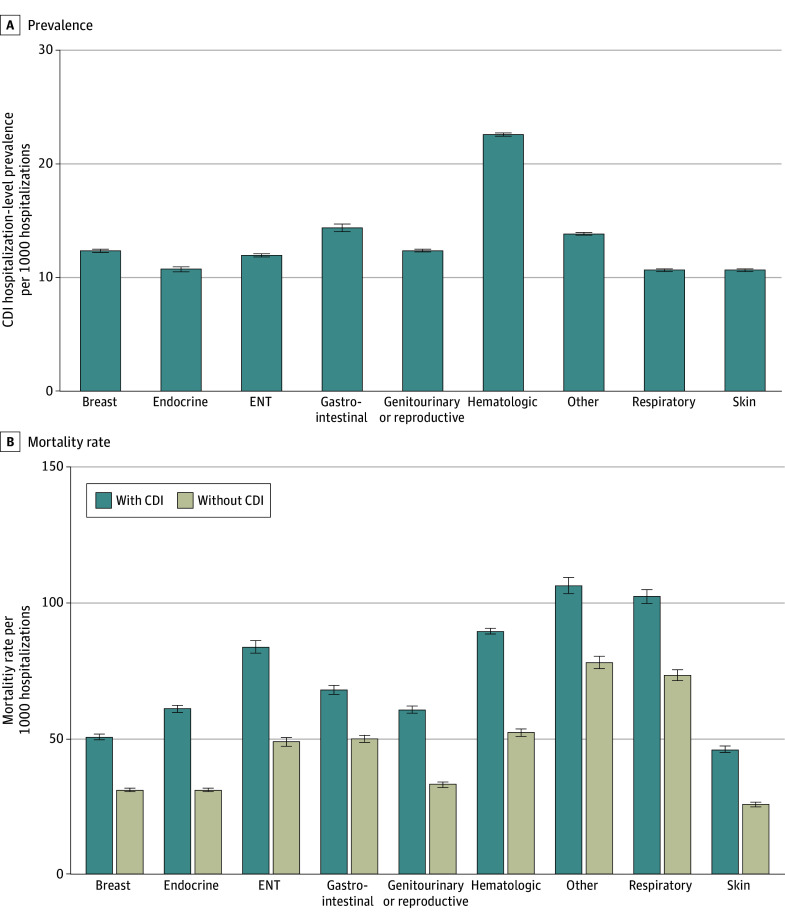
Bar Chart Showing Association of *Clostridioides difficile* Infection (CDI) by Malignant Neoplasm Subtype A: Hospitalization-level prevalence of *Clostridioides difficile* infection per 1000 hospitalizations, by malignant neoplasm subtype (eTable 7 in Supplement 1). B: Mortality rate per 1,000 hospitalizations, by malignant neoplasm subtype and CDI status (CDI first vs no CDI). aOR indicates adjusted odds ratio; ENT, ear, nose, and throat.

Genitourinary and reproductive cancers were the most frequent malignant tumor type among all cancer-related hospitalizations during the study period, while ENT cancers were the least frequent, accounting for 9 334 322 (29.1%) and 335 360 (1.1%) of total admissions, respectively. Among patients with CDI, all-cause mortality ranged from 1905 (4.6%) in those with skin cancers to 11 330 (10.6%) in the other cancers category (Figure 3; eTables 5 and 7 in Supplement 1). The highest rate of KRT was observed in patients with hematologic cancers, with 5515 patients with CDI (5.6%) undergoing RRT during admission (eTable 6 in Supplement 1). Mechanical ventilation was most frequently required in patients with ENT cancers (1015 of those with CDI [13.7%]), who also had the highest vasopressor support rates (255 patients [3.4%]). Across each malignant tumor subgroup, CDI was associated with an increased rate of all-cause mortality, KRT, mechanical ventilation, and vasopressor support, even after adjusting for relevant confounders, including age, sex, race, comorbidities, cancer type, insurance status, and hospital characteristics.

## Discussion

In this nationally representative cross-sectional study of over 32 million US hospitalizations between 2016 and 2022, we found that CDI was associated with significantly worse clinical outcomes and greater health care utilization among patients with malignant tumor–related admissions. Among hospitalized patients with cancer, CDI was independently associated with higher all-cause in-hospital mortality, KRT utilization, mechanical ventilation, and vasopressor support.

The evolving epidemiology and clinical impact of CDI among hospitalized patients with cancer have been well described in prior studies. Luo et al,^14^ analyzing NIS data from 2005 to 2011, reported a 16.7% relative increase in CDI incidence among hospitalized leukemia patients over the study period with an overall incidence of 3.4%. Similarly, using the National Hospital Discharge Survey (NHDS) from 2001 to 2010, Delgado et al^13^ found an overall CDI incidence of 8.6 per 1000 cancer-related hospitalizations with rates peaking at 17.2 per 1000 discharges in 2008. Although prior studies reported increasing CDI incidence among hospitalized patients with cancer through 2011, our analysis of nationwide data from 2016 to 2022 shows a steady decline in CDI rates, from 17.4 to 11.4 cases per 1000 admissions, with an overall hospitalization-level prevalence of 1.4%. This difference may be partly related to differences in patient populations, as the NHDS included only short stay (fewer than 30 days), nonfederal hospitals, which may capture different CDI risk dynamics. While our study focused on malignant tumor–related hospitalizations, national CDI estimates for noncancer admissions during similar periods are substantially lower, typically 5 to 11 cases per 10 000 discharges, which suggests that CDI prevalence remains elevated in oncology patients.^20,21^ This contrast highlights the disproportionate prevalence of CDI among hospitalized patients with cancer, reflecting their increased vulnerability due to immunosuppression, antimicrobial exposure, and frequent health care encounters.

Consistent with earlier reports from the NHDS, our study confirms that CDI is significantly associated with increased in-hospital mortality among patients with cancer. In earlier studies, Gupta et al^12^ reported a mortality rate of 9.1% among patients with cancer with CDI compared with 6.3% in those without CDI, while Delgado et al^13^ similarly observed increased mortality of 9.4% among cancer-related hospitalizations complicated by CDI. In our analysis, the mortality rate among patients with cancer with CDI was 7.3% among patients with cancer with CDI vs 4.5% without CDI, with an aOR of 1.62 (95% CI, 1.58-1.67).

Notably, when stratified by malignant tumor type, the highest mortality rates were observed among patients with respiratory tumors (10.2%) and hematologic cancers (8.9%). These findings are consistent, although at lower absolute rates, with prior reports by Larrainzar-Coghen et al,^22^ who observed a 30-day mortality rate of 19.2% among patients with hematologic cancers, and Luo et al,^14^ 17% higher odds of mortality among leukemia patients (OR, 1.17; 95% CI, 1.13-1.22). Similarly, Selvey et al,^23^ in an Australian study, and Larrainzar-Coghen^22^ found that CDI was associated with significantly higher mortality across both hematologic and solid-organ cancers, and Guddati et al^24^ reported increased mortality among hematopoietic cell transplant recipients with CDI, particularly in those with graft-vs-host disease. The lower all-cause and cancer subtype-specific mortality rates observed in our study, compared with prior reports, likely reflect improvements in early CDI detection and infection control practices during the more contemporary study period (2016-2022).

In addition to variation by malignant tumor type, we observed notable regional differences in CDI-associated hospitalization-level prevalence and mortality among hospitalized oncologic patients with mortality rates ranging from 6.4% in the Midwest to 8.5% in the Northeast. Although our study focused specifically on patients with oncologic-related admissions, prior studies examining broader hospitalized populations have also reported regional disparities. Argamany et al,^25^ using the US NHDS from 2001 to 2010, found that CDI-related mortality was highest in the Midwest (7.3%) and lowest in the West (6.2%) among all hospitalized patients. These patterns may reflect differences in patient comorbidities, infection control practices, health care resources, and access to advanced oncologic care. Further research is needed to clarify the drivers of regional disparities in CDI outcomes among patients with cancer.

The adverse outcomes observed among patients with cancer with CDI may be related to a convergence of factors, including immunosuppression, chemotherapy-induced mucosal injury, neutropenia, and antibiotic exposures, all of which compromise gut integrity and host defenses.^6,7,8,9,10,26^ These mechanisms not only drive higher mortality but are also associated with prolonged hospital stays and increased health care costs. CDI was associated with 4.8 additional hospital days and $11 100 in excess in cost per admission compared with patients without CDI. These differences correspond to a 76% increase in length of stay and a 46% increase in total hospitalization cost, consistent across malignant tumor subtypes. While CDI was associated with significantly higher total hospitalization costs, the daily cost of hospitalization was not increased among patients with CDI, suggesting that the excess financial impact may be largely related to prolonged LOS rather than more intensive daily resource use. Prior studies similarly reported that CDI prolonged hospitalizations by 5.7 days and increased rates of discharge to care facilities (27.3% vs 15.4%).^12,13^

Future studies are needed to elucidate the mechanisms that underlie the increased susceptibility and adverse outcomes of CDI in patients with cancer and to develop targeted strategies to diagnose and mitigate this risk. Although our analysis did not examine CDI treatment patterns, the findings raise important concerns about whether current management algorithms, largely developed in populations without cancer, are appropriate for oncology patients. For instance, recent evidence suggests that fidaxomicin, now recommended as first-line therapy, was often underutilized in patients with cancer during the study period, potentially contributing to suboptimal outcomes.^27^ In light of this, innovative therapies such as conventional fecal microbiota transplantation, shown to be safe even in patients with hematologic cancers, warrant further evaluation in cancer-specific settings.^28,29^ Moreover, questions remain as to whether higher mortality may be related to hospital-acquired infections and whether infection control strategies, such as dedicated oncology wards, could reduce transmission.^30^ Ultimately, these considerations highlight the need for cancer-adapted treatment pathways.

### Strengths and Limitations

This study has several strengths. It represents, to our knowledge, the largest and most recent nationwide analysis evaluating the clinical and economic outcomes of CDI among hospitalized patients with cancer using the NIS database. The large sample size and diversity of hospitals included enhance the generalizability of our findings across different regions and hospital types in the US. Importantly, this is the first study we know of to assess regional variation in CDI hospitalization-level prevalence and mortality rates among patients with cancer using the NIS, providing novel insights into geographic differences in outcomes.

Several limitations should be considered. First, this was a retrospective, cross-sectional analysis of a discharge-level administrative database, which permits assessment of associations but precludes causal inference. CDI was identified using *ICD-10* diagnosis codes, which may lead to exposure misclassification and does not distinguish between recurrent vs first episodes or health care-associated vs community-acquired cases. Our estimates reflect the prevalence of CDI among hospital discharges rather than incidence in the at-risk cancer population. Important clinical details, such as symptoms, illness severity, CDI testing modality, chemotherapy regimens, hematopoietic stem cell transplant status, graft-vs-host disease complications, and medication exposures, are not captured and likely contribute to residual confounding. Because the NIS does not include longitudinal follow-up or exact dates of diagnoses and procedures, we were unable to determine the timing of CDI onset relative to clinical deterioration, organ support, or death. Consequently, time-to-event analyses and modeling of time at risk were not possible. We were also unable to assess CDI history or account for risk factors. As the NIS captures discrete hospitalizations without patient-level linkage, it is likely that some patients contributed multiple admissions. Additionally, we could not adjust for secular trends in cancer care or evolving CDI epidemiology over the study period.

## Conclusions

In this cross-sectional study of hospitalized patients with cancer in the US, CDI was associated with higher in-hospital mortality and greater health care resource utilization across all malignant tumor types and regions. These findings underscore the urgent need for targeted prevention strategies, early recognition, and optimized management approaches to mitigate the impact of CDI in this vulnerable population.

## Notes

Supplement 1. eTable 1. ICD-10 Diagnostic and Procedural Codes Used to Assess Outcomes in Hospitalizations for Patients With Malignant Neoplasm With and Without Clostridioides difficile eTable 2. Univariable Logistic Regression Analysis Assessing Associations Between *Clostridioides difficile *and Clinical Outcomes in Patients With Malignant Neoplasm eTable 3. Missing Variables in Hospitalizations With Patients With Documented Active Malignant Neoplasm From 2016 to 2022 eTable 4. Clostridioides difficile *Infection Among Hospitalized Patients With Cancer Stratified* by *Year* eTable 5. *Comparison of In-Hospital Clinical Outcomes in Patients With Melanoma and Nonmelanoma Skin Cancer Stratified by the Presence of *Clostridioides difficile eTable 6. Comparison of In-Hospital Clinical Outcomes in Subtypes of Malignant Tumor–Related Hospitalizations Stratified by the Presence of *Clostridioides difficile* eTable 7. Hospitalization-Level Prevalence of *Clostridioides difficile* Infection (CDI) by Malignant Neoplasm Subtype (per 1000 Hospitalizations)

Supplement 2. Data Sharing Statement
