# Supplementary material for: Clostridioides difficile Infection Among Hospitalized Patients With Cancer
Source: JAMA Netw Open. 2026 Mar 25;9(3):e262103. doi: 10.1001/jamanetworkopen.2026.2103 (PMC13019233; doi:10.1001/jamanetworkopen.2026.2103)
Supplement: Supplement 1. — eTable 1. ICD-10 Diagnostic and Procedural Codes Used to Assess Outcomes in Hospitalizations for Patients With Malignant Neoplasm With and Without Clostridioides difficile eTable 2. Univariable Logistic Regression Analysis Assessing Associations Between Clostridioides difficile and Clinical Outcomes in Patients With Malignant Neoplasm eTable 3. Missing Variables in Hospitalizations With Patients With Documented Active Malignant Neoplasm From 2016 to 2022 eTable 4. Clostridioides difficile Infection Among Hospitalized Patients With Cancer Stratified by Year eTable 5. Comparison of In-Hospital Clinical Outcomes in Patients With Melanoma and Nonmelanoma Skin Cancer Stratified by the Presence of Clostridioides difficile eTable 6. Comparison of In-Hospital Clinical Outcomes in Subtypes of Malignant Tumor–Related Hospitalizations Stratified by the Presence of Clostridioides difficile eTable 7. Hospitalization-Level Prevalence of Clostridioides difficile Infection (CDI) by Malignant Neoplasm Subtype (per 1000 Hospitalizations) [file jamanetwopen-e262103-s001.pdf]

Supplemental Online Content

Roldan GA, Goble S, Davie T, et al. National estimates of *Clostridioides difficile* infection and outcomes in US cancer hospitalizations. *JAMA Netw Open*. 2026;9(3):e262103. doi:10.1001/jamanetworkopen.2026.2103

eTable 1. ICD-10 Diagnostic and Procedural Codes Used to Assess Outcomes in Hospitalizations for Patients With Malignant Neoplasm With and Without Clostridioides difficile
eTable 2. Univariable Logistic Regression Analysis Assessing Associations Between *Clostridioides difficile* and Clinical Outcomes in Patients With Malignant Neoplasm
eTable 3. Missing Variables in Hospitalizations With Patients With Documented Active Malignant Neoplasm From 2016 to 2022
eTable 4. *Clostridioides difficile* Infection Among Hospitalized Patients With Cancer Stratified by Year
eTable 5. Comparison of In-Hospital Clinical Outcomes in Patients With Melanoma and Nonmelanoma Skin Cancer Stratified by the Presence of *Clostridioides difficile*
eTable 6. Comparison of In-Hospital Clinical Outcomes in Subtypes of Malignant Tumor–Related Hospitalizations Stratified by the Presence of *Clostridioides difficile*
eTable 7. Hospitalization-Level Prevalence of *Clostridioides difficile* Infection (CDI) by Malignant Neoplasm Subtype (per 1000 Hospitalizations)

This supplemental material has been provided by the authors to give readers additional information about their work.

© 2026 Roldan GA et al. *JAMA Network Open*.

eTable 1. ICD-10 Diagnostic and Procedural Codes Used to Assess Outcomes in Hospitalizations for Patients With Malignancy With and Without *Clostridioides difficile*

Clostridioides difficile	A047*
Hematologic cancer	C8100, C8102, C8103, C8104, C8105, C8106, C8107, C8108, C8109, C8110, C8111, C8112, C8113, C8114, C8115, C8116, C8117, C8118, C8119, C8120, C8121, C8122, C8123, C8124, C8125, C8126, C8127, C8128, C8129, C8130, C8131, C8132, C8133, C8134, C8135, C8136, C8137, C8138, C8139, C8140, C8141, C8142, C8143, C8144, C8145, C8146, C8147, C8148, C8149, C8170, C8171, C8172, C8173, C8174, C8175, C8176, C8177, C8178, C8179, C8190, C8191, C8192, C8193, C8194, C8195, C8196, C8197, C8198, C8199, C8200, C8201, C8202, C8203, C8204, C8205, C8206, C8207, C8208, C8209, C8210, C8211, C8212, C8213, C8214, C8215, C8216, C8217, C8218, C8219, C8220, C8221, C8222, C8223, C8224, C8225, C8226, C8227, C8228, C8229, C8230, C8231, C8232, C8233, C8234, C8235, C9400, C9401, C9402, C9420, C9421, C9422, C9430, C9431, C9432, C9440, C9441, C9442, C946, C9480, C9481, C9482, C9500, C9501, C9502, C9510, C9511, C9512, C9590, C9591, C9592, Z856, C9000, C9001, C9002, C9010, C9011, C9012, C9020, C9021, C9022, C9030, C9031, C9032, D460, D461, D4620, D4621, D4622, D464, D469, D46A, D46B, D46C, D46Z, C8585, C8586, C8587, C8588, C8589, C8590, C8591,

© 2026 Roldan GA et al. *JAMA Network Open*.

	C8592, C8593, C8594, C8595, C8596, C8597, C8598, C8599, C860, C861, C862, C863, C864, C865, C866, C880, C882, C883, C884, C888, C889, C960, C962, C965, C966, C969, C96Z, Z8571, Z8572, Z8579, C9100, C9101, C9102, C9110, C9111, C9112, C9130, C9131, C9132, C9140, C9141, C9142, C9150, C9151, C9152, C9160, C9161, C9162, C9190, C9191, C9192, C91A0, C91A1, C91A2, C91Z0, C91Z1, C9200, C9201, C9202, C9210, C9211, C9212, C9220, C9221, C9222, C9240, C9241, C9242, C9250, C9251, C9252, C9260, C9261, C9262, C9290, C9291, C9292, C92A0, C92A1, C92A2, C92Z0, C92Z1, C9300, C9301, C9302, C9310, C9311, C9312, C9330, C9331, C9332, C9390, C9391, C9392, C93Z0, C93Z1, C93Z2
Ear, nose, and throat cancer	C000, C001, C002, C003, C004, C005, C006, C008, C009, C01, C020, C021, C022, C023, C024, C028, C029, C030, C031, C039, C040, C041, C048, C049, C050, C051, C052, C058, C059, C060, C061, C062, C0680, C0689, C069, C07, C080, C081, C089, C090, C091, C098, C099, C100, C101, C102, C103, C104, C108, C109, C110, C111, C112, C113, C118, C119, C12, C130, C131, C132, C133, C138, C139, C140, C142, C148, C300, C301, C310, C311, C312, C313, C318, C319, C320, C321, C322, C323, C328, C329, C6900,

© 2026 Roldan GA et al. *JAMA Network Open*.

	C6901, C6902, C6910, C6911, C6912, C6920, C6921, C6922, C6930, C6931, C6932, C6940, C6941, C6942, C6950, C6951, C6952, C6960, C6961, C6962, C6980, C6981, C6982, C6990, C6991, C6992, C760, D0000, D0001, D0002, D0003, D0004, D0005, D0006, D0007, D0008, D020, D0920, D0921, D0922
Gastrointestinal cancer	C153, C154, C155, C158, C159, C49A1, D001, C160, C161, C162, C163, C164, C165, C166, C168, C169, C49A2, D002, Z85020, Z85028, C170, C171, C172, C173, C178, C179, C49A3, D0149, Z85060, Z85068, C180, C181, C182, C183, C184, C185, C186, C187, C188, C189, C19, C20, C260, C49A4, C49A5, D010, D011, D012, D0140, C7A020, C7A021, C7A022, C7A023, C7A024, C7A025, C7A026, C7A029, Z85030, Z85038, Z85040, Z85048, C220, C222, C223, C224, C227, C228, C229, D015, Z8505, C210, C211, C212, C218, D013, C221, D015, C23, C240, C241, C248, C249, C261, C269, C480, C481, C482, C488, C49A0, C49A9, D017, D019, C250, C251, C252, C253, C254, C257, C258, C259
Respiratory cancer	C33, C34, C3400, C3401, C3402, C3410, C3411, C3412, C342, C3430, C3431, C3432, C3480, C3481, C3482, C3490, C3491, C3492, C384, C39, C390, D021, D0220, D0221, D0222, D023, D024, Z85118, Z8512, Z8520

© 2026 Roldan GA et al. *JAMA Network Open*.

Skin cancer	C430, C4310, C4311, C43111, C43112, C4312, C43121, C43122, C4320, C4321, C4322, C4330, C4331, C4339, C434, C4351, C4352, C4359, C4360, C4361, C4362, C4370, C4371, C4372, C438, C439, C4400, C4401, C4402, C4409, C44101, C44102, C441021, C441022, C44109, C441091, C441092, C44111, C44112, C441121, C441122, C44119, C441191, C441192, C44121, C44122, C441221, C441222, C44129, C441291, C441292, C44131, C441321, C441322, C441391, C441392, C44191, C44192, C44199, C441921, C441922, C441991, C441992, C44201, C44202, C44209, C44211, C44212, C44219, C44221, C44222, C44229, C44291, C44292, C44299, C44300, C44301, C44309, C44310, C44311, C44319, C44320, C44321, C44329, C44390, C44391, C44399, C4440, C4441, C4442, C4449, C44500, C44501, C44509, C44510, C44511, C44519, C44520, C44521, C44529, C44590, C44591, C44599, C44601, C44602, C44609, C44611, C44612, C44619, C44621, C44622, C44629, C44691, C44692, C44699, C44701, C44702, C44709, C44711, C44712, C44719, C44721, C44722, C44729, C44791, C44792, C44799, C4480, C4481, C4482, C4489, C4490, C4491, C4492, C4499, C4A0, C4A10, C4A11, C4A111, C4A112, C4A12, C4A121, C4A122, C4A20, C4A21, C4A22, C4A30, C4A31, C4A39, C4A4, C4A51, C4A52, C4A59, C4A60, C4A61, C4A62, C4A70, C4A71, C4A72, C4A8, C4A9,

© 2026 Roldan GA et al. *JAMA Network Open*.

	D030, D0310, D0311, D03111, D03112, D0312, D03121, D03122, D0320, D0321, D0322, D0330, D0339, D034, D0351, D0352, D0359, D0360, D0361, D0362, D0370, D0371, D0372, D038, D039, D040, D0410, D0411, D04111, D04112, D0412, D04121, D04122, D0420, D0421, D0422, D0430, D0439, D044, D045, D0460, D0461, D0462, D0470, D0471, D0472, D048, D049, Z85820, Z85828
Breast cancer	C50011, C50012, C50019, C50021, C50022, C50029, C50111, C50112, C50119, C50121, C50122, C50129, C50211, C50212, C50219, C50221, C50222, C50229, C50311, C50312, C50319, C50321, C50322, C50329, C50411, C50412, C50419, C50421, C50422, C50429, C50511, C50512, C50519, C50521, C50522, C50529, C50611, C50612, C50619, C50621, C50622, C50629, C50811, C50812, C50819, C50821, C50822, C50829, C50911, C50912, C50919, C50921, C50922, C50929, D0500, D0501, D0502, D0510, D0511, D0512, D0580, D0581, D0582, D0590, D0591, D0592, Z853

© 2026 Roldan GA et al. *JAMA Network Open*.

Genitourinary and reproductive cancer	C641, C642, C649, C651, C652, C659, C661, C662, C669, C670, C671, C672, C673, C674, C675, C676, C677, C678, C679, C680, C681, C688, C689, D090, D0910, D0919, Z8551, C7A093, Z8550, Z8551, Z85520, Z85528, Z8553, Z8554, Z8559, C540, C542, C543, C548, C549, C55, Z8542, C530, C531, C538, C539, D060, D061, D067, D069, C561, C562, C569, Z8543, C510, C511, C512, C518, C519, C52, C541, C5700, C5701, C5702, C5710, C5711, C5712, C5720, C5721, C5722, C573, C574, C577, C578, C579, C58, D070, D071, D072, D0730, D0739, C61, D075, Z8546, C6200, C6201, C6202, C6210, C6211, C6212, C6290, C6291, C6292, Z8547, C600, C601, C602, C608, C609, C6300, C6301, C6302, C6310, C6311, C6312, C632, C637, C638, C639, D074, D0760, D0761, D0769
Endocrine cancer	D093, E3122, E3123, Z85850, C37, C7400, C7401, C7402, C7410, C7411, C7412, C7490, C7491, C7492, C750, C751, C752, C753, C754, C755, C758, C759, C7A00, C7A010, C7A011, C7A012, C7A019, C7A020, C7A021, C7A022, C7A023, C7A024, C7A025, C7A026, C7A029, C7A090, C7A091, C7A092, C7A093, C7A094, C7A095, C7A096, C7A098, C7A1, C7A8, C7B00, C7B01, C7B02, C7B03, C7B04, C7B09, C7B1, C7B8

© 2026 Roldan GA et al. *JAMA Network Open*.

Other cancer	Secondary neoplasms (e.g., liver, brain, bone, lung) C770-C779, C7800-C7802, C781-C789, C7900-C799<br>Connective and soft tissue neoplasms C490-C499<br>Primary malignant neoplasms of bone C4000-C4092, C410-C419<br>Neuroendocrine tumors C7A090<br>Brain and spinal cord tumors C700-C729<br>Lymphoid neoplasms not otherwise categorized C460-C469<br>Unspecified thoracic, hematopoietic, and ill-defined neoplasms C380-C383, C388, C9622, C964, C96A<br>Personal history of malignant neoplasms Z85841, Z85848<br>Myelodysplastic syndromes D46Z<br>Cervical and other carcinoma in situ D098<br>Neoplasms of peripheral nerves C470-C475, C476, C478, C479
Ulcerative colitis	K51*
Crohn's	K50*
Solid organ transplant history	Z941, Z942, Z944, Z9482, Z940
HIV	B20.0, B20.1, B20.2, B20.3, B20.4, B20.5, B20.6, B20.7, B20.8, B20.9, B21.0. B21.1, B21.2, B21.3, B21.7, B21.8, B21.9, B22.0,

© 2026 Roldan GA et al. *JAMA Network Open*.

	B22.1, B22.2, B22.7, B23.0, B23.1, B23.2, B23.8, B20, B24, Z21
Cirrhosis	K7030, K7031, K7460, K7469, K743, K744, K745, K717
Chronic kidney disease	N18, N181, N182, N183, N184, N185, N186, N187, N188, N189, N19, R944
Renal replacement therapy	5A1D00Z, 5A1D60Z, 5A1D70Z, 5A1D80Z, 5A1D90Z
Mechanical ventilation	0BH17EZ, 0BH18EZ, 5A1935Z, 5A1945Z, 5A1955Z
Vasopressor support	3E030XZ, 3E033XZ, 3E040XZ, 3E043XZ, 3E050XZ, 3E053XZ, 3E060XZ, 3E063XZ

© 2026 Roldan GA et al. *JAMA Network Open*.

eTable 2. Univariable Logistic Regression Analysis Assessing Associations Between *Clostridioides difficile* and Clinical Outcomes in Patients With Malignant Neoplasm

Clinical	Unadjusted odds ratio (95% CI)	*P*-value
Mortality	1.73 (1.69-1.78)	<0.001
Renal replacement therapy	2.27 (2.20-2.35)	<0.001
Mechanical ventilation	2.02 (1.97-2.08)	<0.001
Vasopressor support	2.31 (2.18-2.45)	<0.001

© 2026 Roldan GA et al. *JAMA Network Open*.

eTable 3. Missing Variables in Hospitalizations With Patients With Documented Active Malignant Neoplasm From 2016 to 2022

Variable	Total hospitalizations with missing data	Proportion
Age	725	0.0%
Sex	8,205	0.0%
Race	827,460	2.6%
Hospital bed size	0	0.0%
Hospital region	0	0.0%
Income quartile	477,030	1.5%
Primary payer	868,009	2.7%
Length of stay	1,135	0.0%
Cost	246,630	0.8%

© 2026 Roldan GA et al. *JAMA Network Open*.

eTable 4. *Clostridioides difficile* Infection Among Hospitalized Patients With Cancer Stratified by Year

Year	Total hospitalizations with documented *Clostridioides difficile* infection	Cases per 1,000 admission
2016	81,165	17.4
2017	75,155	15.6
2018	71,250	14.7
2019	65,135	13.1
2020	53,354	12.0
2021	52,335	11.5
2022	51,970	11.4

© 2026 Roldan GA et al. *JAMA Network Open*.

eTable 5. Comparison of In-Hospital Clinical Outcomes in Patients With Melanoma and Nonmelanoma Skin Cancer Stratified by the Presence of *Clostridioides difficile*

	Clostridium difficile	No *Clostridium difficile*	Adjusted odds ratio (95% CI)	*P*-value
Melanoma	38,845 (1.1%)	3,645,164 (98.9%)	-	-
Mortality	4.5%	2.5%	1.77 (1.57-1.98)	<0.001
Renal replacement therapy	3.5%	1.4%	2.31 (2.02-2.64)	<0.001
Mechanical ventilation	4.0%	2.1%	1.92 (1.71-2.16)	<0.001
Vasopressor support	1.6%	0.8%	2.16 (1.81-2.59)	<0.001
Colonoscopy	3.4%	1.6%	2.10 (1.85-2.38)	<0.001
Non-melanoma	2,650 (1.3%)	208,910 (98.7%)	-	-
Mortality	6.2%	4.0%	1.55 (1.07-2.24)	0.02
Renal replacement therapy	6.4%	2.1%	2.94 (2.02-4.28)	<0.001
Mechanical ventilation	6.7%	3.4%	1.97 (1.38-2.81)	<0.001
Vasopressor support	2.5%	1.1%	2.15 (1.21-3.82)	0.01
Colonoscopy	1.3%	1.3%	0.99 (0.47-2.11)	0.98

© 2026 Roldan GA et al. *JAMA Network Open*.

eTable 6. Comparison of In-Hospital Clinical Outcomes in Subtypes of Malignant Tumor–Related Hospitalizations Stratified by the Presence of *Clostridioides difficile*

© 2026 Roldan GA et al. *JAMA Network Open*.

	Clostridioides difficile (n = 450,360)	No *Clostridioides difficile* (n = 31,633,311)		
**Clinical Outcome**	**Rate per 1,000 hospitalizations**	**Rate per 1,000 hospitalizations**	**Adjusted* Odds Ratio (95% CI)**	***P*-value**
**Hematologic**				
Mortality	88.9	52.2	1.86 (1.76-1.96)	<0.001
Renal replacement therapy	56.2	31.4	1.82 (1.70-1.95)	<0.001
Mechanical ventilation	78.4	41.9	1.93 (1.82-2.04)	<0.001
Vasopressor support	30.9	14.4	2.10 (1.93-2.30)	<0.001
**Ear, nose and throat**				
Mortality	83.7	48.8	1.73 (1.41-2.11)	<0.001
Renal replacement therapy	20.9	8.7	2.20 (1.50-3.24)	<0.001
Mechanical ventilation	137.0	76.4	1.88 (1.61-2.20)	<0.001

© 2026 Roldan GA et al. *JAMA Network Open*.

Vasopressor support	34.4	15.7	2.14 (1.58-2.89)	<0.001
**Gastrointestinal**				
Mortality	67.9	49.8	1.36 (1.27-1.45)	<0.001
Renal replacement therapy	38.6	17.7	2.14 (1.98-2.32)	<0.001
Mechanical ventilation	60.7	33.7	1.83 (1.72-1.95)	<0.001
Vasopressor support	23.9	12.7	1.85 (1.66-2.06)	<0.001
**Respiratory**				
Mortality	102.3	73.3	1.43 (1.32-1.54)	<0.001
Renal replacement therapy	27.7	11.4	2.33 (2.01-2.69)	<0.001
Mechanical ventilation	86.3	51.8	1.75 (1.62-1.90)	<0.001
Vasopressor support	28.1	12.3	2.34 (2.03-2.68)	<0.001
**Skin**				

© 2026 Roldan GA et al. *JAMA Network Open*.

Mortality	46.0	25.8	1.75 (1.56-1.95)	<0.001
Renal replacement therapy	37.1	14.6	2.38 (2.10-2.70)	<0.001
Mechanical ventilation	41.9	21.8	1.93 (1.72-2.16)	<0.001
Vasopressor support	16.6	7.9	2.17 (1.83-2.58)	<0.001
**Breast**				
Mortality	50.6	31.1	1.56 (1.43-1.70)	<0.001
Renal replacement therapy	29.3	14.5	1.92 (1.71-2.16)	<0.001
Mechanical ventilation	45.4	25.8	1.73 (1.58-1.89)	<0.001
Vasopressor support	16.4	7.7	2.05 (1.76-2.38)	<0.001
**Genitourinary and reproductive**				
Mortality	60.6	33.1	1.71 (1.61-1.81)	<0.001

© 2026 Roldan GA et al. *JAMA Network Open*.

Renal replacement therapy	51.1	25.7	1.85 (1.74-1.97)	<0.001
Mechanical ventilation	54.6	27.0	1.99 (1.87-2.11)	<0.001
Vasopressor support	20.8	9.4	2.13 (1.92-2.36)	<0.001
**Endocrine**				
Mortality	61.0	32.1	1.84 (1.50-2.24)	<0.001
Renal replacement therapy	50.9	18.4	2.51 (2.00-3.15)	<0.001
Mechanical ventilation	75.3	32.3	2.30 (1.93-2.74)	<0.001
Vasopressor support	23.9	11.2	2.10 (1.53-2.88)	<0.001
**Other cancer**				
Mortality	106.3	78.0	1.37 (1.31-1.44)	<0.001
Renal replacement therapy	33.6	13.1	2.35 (2.16-2.56)	<0.001

© 2026 Roldan GA et al. *JAMA Network Open*.

Mechanical ventilation	76.3	43.4	1.81 (1.71-1.91)	<0.001
Vasopressor support	31.6	15.5	2.03 (1.85-2.23)	<0.001

* Adjusted for age, sex, race, income quartile, Charlson Comorbidity index, primary payer, hospital bed size, and hospital region

© 2026 Roldan GA et al. *JAMA Network Open*.

eTable 7. Hospitalization-Level Prevalence of *Clostridioides difficile* Infection (CDI) by Malignant Neoplasm Subtype (per 1000 Hospitalizations)

Malignancy Subtype	CDI Prevalence (per 1,000)
Breast	12.4
Endocrine	10.8
ENT	12.0
Gastrointestinal	14.4
Genitourinary/Reproductive	12.4
Hematologic	22.6
Other	13.9
Respiratory	10.7
Skin	10.7

© 2026 Roldan GA et al. *JAMA Network Open*.
