# Supplementary material for: Clostridioides difficile Infection Among Hospitalized Patients With Cancer
Source: JAMA Netw Open. 2026 Mar 25;9(3):e262103. doi: 10.1001/jamanetworkopen.2026.2103 (PMC13019233; doi:10.1001/jamanetworkopen.2026.2103)
Supplement: Supplement 2. — Data Sharing Statement [file jamanetwopen-e262103-s002.pdf]

Data Sharing Statement

Roldan. Clostridioides difficile Infection and Associated Outcomes in Cancer Hospitalizations. *JAMA Netw Open*. Published March 25, 2026. doi:10.1001/jamanetworkopen.2026.2103

Data
Data available: No

Additional Information
Explanation for why data not available: The data used in this study were derived from a nationally representative, de-identified dataset licensed from the Healthcare Cost and Utilization Project (HCUP-NIS). Due to data use agreement restrictions and licensing terms, we are not permitted to share the raw dataset. Interested researchers may obtain access independently through HCUP at https://www.hcup-us.ahrq.gov/
